# Hepatic hydrothorax does not increase the risk of death after transjugular intrahepatic portosystemic shunt in cirrhosis patients

**DOI:** 10.1007/s00330-022-09357-3

**Published:** 2022-12-28

**Authors:** Xiaoyuan Gou, Wenyuan Jia, Chuangye He, Xulong Yuan, Jing Niu, Jiao Xu, Na Han, Ying Zhu, Wenlan Wang, Jun Tie

**Affiliations:** 1 National Clinical Research Center for Digestive Diseases and Xijing Hospital of Digestive Diseases, Air Force Medical University, Xi’an, 710032 Shaanxi China; 2 Department of Aerospace Hygiene, School of Aerospace Medicine, Air Force Medical University, Xi’an, 710032 Shaanxi China

**Keywords:** Hydrothorax, Prognosis, Hypertension, portal, Portasystemic shunt, transjugular intrahepatic

## Abstract

**Objectives:**

Hepatic hydrothorax (HH) is a predictor of poor survival in cirrhosis patients. However, whether HH increases the mortality risk of cirrhosis patients treated with transjugular intrahepatic portosystemic shunt (TIPS) is unknown. Our objective was to evaluate the influence of HH on the survival of cirrhosis patients after TIPS.

**Methods:**

Cirrhosis patients with portal hypertension complications were selected from a prospective database of consecutive patients treated with TIPS in Xijing Hospital from January 2015 to June 2021. Cirrhosis patients with HH were treated as the experimental group. A control group of cirrhosis patients without HH was created using propensity score matching. Survival after TIPS and the related risk factors were analysed.

**Results:**

There were 1292 cirrhosis patients with portal hypertension complications treated with TIPS, among whom 255 patients had HH. Compared with patients without HH, patients with HH had worse liver function (MELD, 12 vs. 10, *p* < 0.001), but no difference in survival after TIPS was observed. After propensity score matching, 243 patients with HH and 243 patients without HH were enrolled. There was no difference in cumulative survival between patients with and without HH. Cox regression analysis showed that HH was not associated with survival after TIPS, and main portal vein thrombosis (> 50%) was a prognostic factor of long-term survival after TIPS in cirrhosis patients (hazard ratio, 1.386; 95% CI, 1.030–1.865, *p* = 0.031).

**Conclusion:**

Hepatic hydrothorax does not increase the risk of death after TIPS in cirrhosis patients.

**Key Points:**

• *Hepatic hydrothorax is a decompensated event of cirrhosis and increases the risk of death.*

• *Hepatic hydrothorax is associated with worse liver function.*

• *Hepatic hydrothorax does not increase the mortality of cirrhosis treated with TIPS.*

**Supplementary Information:**

The online version contains supplementary material available at 10.1007/s00330-022-09357-3.

## Introduction

Hepatic hydrothorax (HH) is defined as a pleural effusion, usually over 500 ml, in cirrhosis patients with portal hypertension but without cardiopulmonary disease [1]. The incidence of HH in patients with cirrhosis is approximately 5–10% [2–4]. The clinical manifestations of HH depend on the amount of pleural effusion, rate of development, and patient tolerance. When the pleural effusion volume is small, there may be no symptoms. If the pleural fluid exceeds 1000 ml, chest tightness, shortness of breath, cough, dyspnoea, and even respiratory failure may occur [5, 6].

Previous studies have shown that HH is a decompensated event of cirrhosis and is associated with poor prognosis [6–12]. Osman KT et al [12] studied the effect of refractory HH and ascites on survival. The results showed that the 1-year mortality of the HH group was 51.06% and that of the refractory ascites group was 19.15%. The median survival time of patients with refractory HH was 4.87 months, and the median survival time of patients with refractory ascites was over 1 year. Not only do patients with refractory HH have a high risk of death, but those with nonrefractory HH who are effectively treated with diuretics and/or thoracentesis also have poor survival. A retrospective case–control study found that the 5-year survival rate was 60% in the HH group and 100% in the control group [8]. These studies showed a higher mortality rate in patients with HH.

Transjugular intrahepatic portosystemic shunt (TIPS) is a crucial therapeutic option for the complications of portal hypertension with cirrhosis, including gastrointestinal bleeding, ascites, and hepatic hydrothorax [5, 13]. The complete and partial response rates of refractory HH were 59% to 81% [14, 15]. However, the 1-year mortality rate after TIPS was more than 50% [16, 17]. TIPS should be used carefully in patients with refractory HH [18]. Previous studies have found that TIPS does not improve the survival of refractory ascites [19], but can improve the survival of recurrent ascites [20]. For patients with variceal bleeding and nonrefractory ascites, TIPS can effectively control bleeding and ascites, and also improve survival [21]. However, whether TIPS improves survival in patients with variceal bleeding and nonrefractory HH is still unclear. Thus, this study aimed to evaluate the effect of HH on the survival of TIPS in cirrhosis patients with portal hypertension complications.

## Methods

### Patients and methods

This was a retrospective case–control study approved by the Medical Ethics Committee of the First Affiliated Hospital of Air Force Medical University (approval number: KY20222200-C-1). Consecutive cirrhosis patients with portal hypertension–related complications treated by TIPS from January 2015 to June 2021 were enrolled. The last patient enrolled was followed up for more than 1 year.

The inclusion criteria for cirrhosis patients with HH were as follows: (1) age older than 18 years; (2) cirrhosis diagnosed by biopsy or imaging; (3) portal hypertension–related complications, such as variceal bleeding and ascites, refractory to medical and/or endoscopic treatment; (4) known symptomatic HH based on chest X-ray and/or computed tomography (CT); and (5) record of at least one postoperative follow-up. The exclusion criteria were as follows: (1) malignant tumours (including primary liver cancer) or other diseases that could shorten the patient’s life span; (2) presence of chest and/or lung infections; (3) presence of TIPS contraindications: New York Heart Association (NYHA) grade ≥ III heart failure, spontaneous bacterial peritonitis, human immunodeficiency virus (HIV) infection, or acquired immunodeficiency syndrome (AIDS)–related diseases; and (4) no follow-up data available.

After the HH patients were screened and included, they were matched to patients without HH based on age, sex, aetiology, model of end-stage liver disease (MELD) score, main portal vein thrombosis, and splenectomy. SPSS (IBM SPSS, version 26) was applied for propensity score matching, and the matching tolerance was 0.01.

### Diagnosis and definitions

Cirrhosis was diagnosed according to the results of liver biopsy or a combination of medical history, aetiology, clinical presentation, and imaging examinations [18].

HH was diagnosed based on the following criteria: (1) cirrhosis patients with portal hypertension had radiographically proven pleural effusion, accompanied by chest tightness, shortness of breath, dyspnoea, hypoxaemia, and even respiratory failure; (2) echocardiography was used to rule out heart failure, and chest CT was used to rule out pulmonary and pleural diseases; and (3) transudative hydrothorax was diagnosed according to Light’s criteria. When patients were treated with diuretics and the pleural fluid/serum albumin ratio was < 0.6, pleural effusion was still diagnosed as HH even though it was considered exudate based on Light’s criteria [1]. Refractory HH was defined as pleural effusion persisting despite successful treatment of ascites [18, 22].

The quantification of HH was performed by using the picture archiving and communication system (PACS)–based volumetric tools [23]. Each patient was measured by 2 senior imaging specialists and averaged. The response of HH to TIPS was assessed according to clinical symptoms or radiographic evidence of hydrothorax post-TIPS [16]. Responses were categorised as complete, partial, or absent. The detailed evaluation criteria were listed in supplementary methods.

### TIPS procedure details and technique

TIPS was mainly performed by Tie Jun and He Chuangye, who had more than 10 years of experience in over 1000 TIPS operations. The main procedure of TIPS was as follows: The femoral artery was punctured by the Seldinger method. Indirect portal vein angiography was performed by selective intubation to the superior mesenteric artery. The internal jugular vein was punctured, an appropriate .035-in. guide wire (Terumo) was introduced into the inferior vena cava via the jugular vein, and the RUPS 100 puncture set (Cook) was advanced over the guide wire into the inferior vena cava. The pressures of the inferior vena cava and hepatic vein were measured. The portal vein branch was punctured from the appropriate hepatic vein or inferior vena cava, confirmed by pushing a small amount of contrast agent. The guide wire was manoeuvred to the splenic vein or distal superior mesenteric vein, the hard guide wires (Cook) were exchanged, the 10 Fr catheter-and-cannula assembly (Cook) was advanced to the main portal vein, the varicose veins were embolised, and the portal vein pressure was measured before and after embolisation. An 8-mm-diameter coated stent (Gore) was implanted, and a 6-mm balloon (BARD) was used for expansion. The pressure on the upper and lower ends of the stent was measured. If the decrease in the portal pressure gradient (PPG) was not ideal, an additional 8-mm balloon (BARD) was used for dilation to reduce the PPG to less than 12 mmHg (16 cmH_2_O) [24] or more than 25% from baseline [25].

### Follow-up and data collection

The following data were collected for all patients: (1) baseline data: case number, age, sex, aetiology of cirrhosis, clinical manifestations, routine blood tests, blood coagulation, blood glucose, serum creatinine, MELD score, biochemical and cytological examination of pleural effusion, chest X-ray and/or chest CT, abdominal B-mode ultrasound, and abdominal contrast-enhanced CT; (2) intraoperative data: PPG before and after stent implantation and operation-related complications; and (3) follow-up data: re-examination at any time due to discomfort. Otherwise, the follow-up visits were carried out regularly at 1, 3, 6, and 12 months after TIPS and every 6 months thereafter. All patients were followed up for life. The recorded data included changes in the main symptoms, HH response to TIPS treatment, stent patency, hepatic encephalopathy (HE), patient survival, blood biochemistry, and MELD score. The baseline and intraoperative data were obtained from medical records, and the follow-up data were obtained from follow-up records.

### Statistical analyses

Quantitative variables are described as the medians (range), and qualitative variables are described as the absolute and relative frequencies. Nonparametric testing was adopted for the medians, chi-square tests were used to compare the frequencies and proportions, and the Kaplan–Meier method was used to analyse overall survival. A Cox proportional hazards regression model was used to analyse the prognostic factors of survival after TIPS in cirrhosis patients. The nonlinear relationships between age, MELD score, and survival were analysed using regression curve estimation. Data analysis was performed using SPSS (IBM SPSS, Version 26, SPSS). All tests were two-sided, and a *p* value < 0.05 was considered statistically significant.

## Results

### Baseline characteristics

**Fig. 1:**
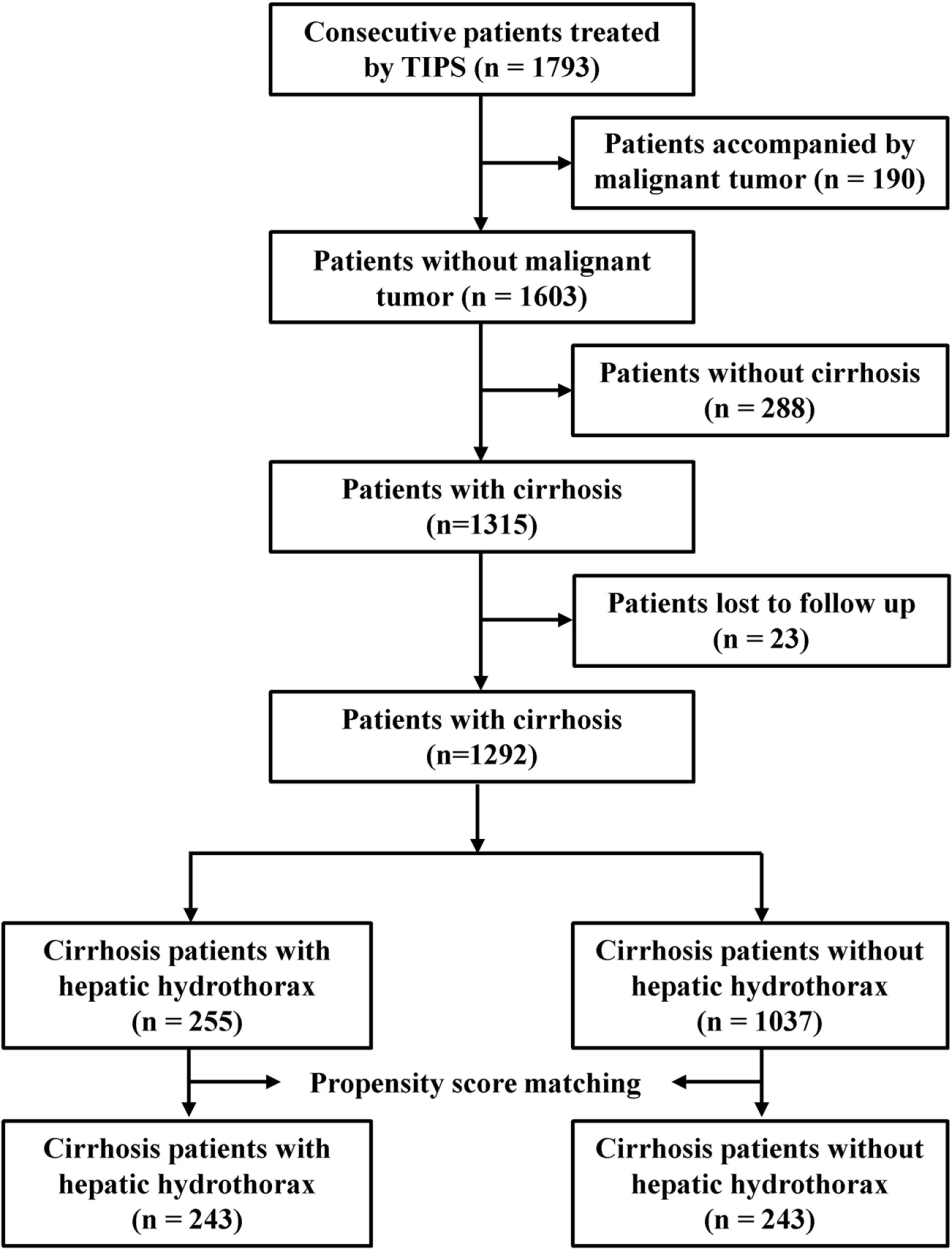
Flowchart of patient selection

From January 2015 to June 2021, 1793 consecutive patients with portal hypertension complications were treated with TIPS. In total, 190 patients with malignant tumours, 288 patients with noncirrhotic portal hypertension, and 23 patients lost to follow-up were excluded. Then, 1292 patients with decompensated cirrhosis who received TIPS were included in this study. There were 255 patients with HH and 1037 patients without HH (Fig. 1). Among all patients, 789 (61.1%) patients were male and 771 (59.7%) patients were infected with hepatitis B virus (HBV). A total of 1116 (91.0%) patients received TIPS due to repeated variceal bleeding, 97 (7.5%) patients received TIPS due to refractory ascites, and 3 (0.2%) patients received TIPS due to refractory HH. A total of 357 (27.6%) patients had main portal vein thrombosis, and the clot obstructed > 50% of the original vessel lumen. A total of 166 (12.8%) patients underwent splenectomy (Table 1).

**Table 1:** Demographic and clinical characteristics of the 1292 patients

Parameter Median (range) or absolute (percentage)	Value
Age (years)	53 (19–89)
Gender	
Male	789 (61.1%)
Female	503 (38.9%)
Aetiology	
HBV	771 (59.7%)
Others	521 (40.3%)
Manifestations	
Variceal bleeding	1176 (91.0%)
Refractory ascites	97 (7.5%)
Refractory hepatic hydrothorax	3 (0.2%)
Others	16 (1.3%)
MELD	11 (0–38)
PVT:MPV > 50%	357 (27.6%)
Splenectomy	166 (12.8%)
Hepatic hydrothorax	255 (19.7%)
Median survival time	1071 (0–2749)

*HBV* hepatitis B virus, *MELD* model of end-stage liver disease, *PVT* portal vein thrombosis, *MPV* main portal vein

### HH is associated with worse liver function, but does not increase the mortality of TIPS

An analysis of the baseline data of patients with and without HH showed that patients with HH had worse liver function (higher MELD score, 12 vs. 10, *p* < 0.001). The patients with HH were treated more often with TIPS for refractory ascites and pleural effusion than patients without HH (Table 2). Furthermore, logistic regression analysis suggested that the MELD score was a risk factor for HH (*p* < 0.001, OR = 2.121, 95% CI, 1.589~2.832) (Table 3). These results suggest that HH is more likely to occur in cirrhotic patients with ascites and poor liver function.

**Table 2:** Comparison between groups based on the presence or absence of hepatic hydrothorax

Parameter Median (range) or absolute (percentage)	HH (255)	No HH (1037)	*p* value
Age (years)	52 (26–85)	53 (19–89)	0.706
Gender			
Male	145 (56.9%)	644 (62.1%)	0.124
Female	110 (43.1%)	393 (37.9%)	
Aetiology			
HBV	146 (57.3%)	625 (60.3%)	0.379
Others	109 (42.7%)	412 (39.7%)	
Manifestations			
Variceal bleeding	219 (85.9%)	957 (92.3%)	0.001^*^
Refractory ascites	31 (12.1%)	66 (6.3%)	0.001^*^
Refractory HH	3 (1.2%)	0 (0%)	0.000^*^
Others	2 (0.8%)	14 (1.4%)	0.464
MELD	12 (0–27)	10 (0–38)	0.000^*^
PVT:MPV > 50%	69 (27.1%)	288 (27.8%)	0.819
Splenectomy	35 (13.7%)	131 (12.6%)	0.640

*HBV* hepatitis B virus, *HH* hepatic hydrothorax, *MELD* model of end-stage liver disease, *PVT* portal vein thrombosis, *MPV* main portal vein

The bold font and asterisks indicate a significant difference

**Table 3:** Logistic regression analysis of factors associated with hepatic hydrothorax

Variable	*p* value	OR	95% CI	
Age (> 53)	0.354	0.867	0.641	1.172
Gender (male)	0.140	0.790	0.578	1.080
Aetiology (HBV)	0.178	0.806	0.589	1.103
MELD (> 11)	0.000^*^	2.121	1.589	2.832
PVT (MPV > 50%)	0.480	0.887	0.635	1.237
Splenectomy	0.251	1.294	0.833	2.011

*HBV* hepatitis B virus, *MELD* model of end-stage liver disease, *PVT* portal vein thrombosis, *MPV* main portal vein

The bold font and asterisks indicate a significant difference

**Fig. 2:**
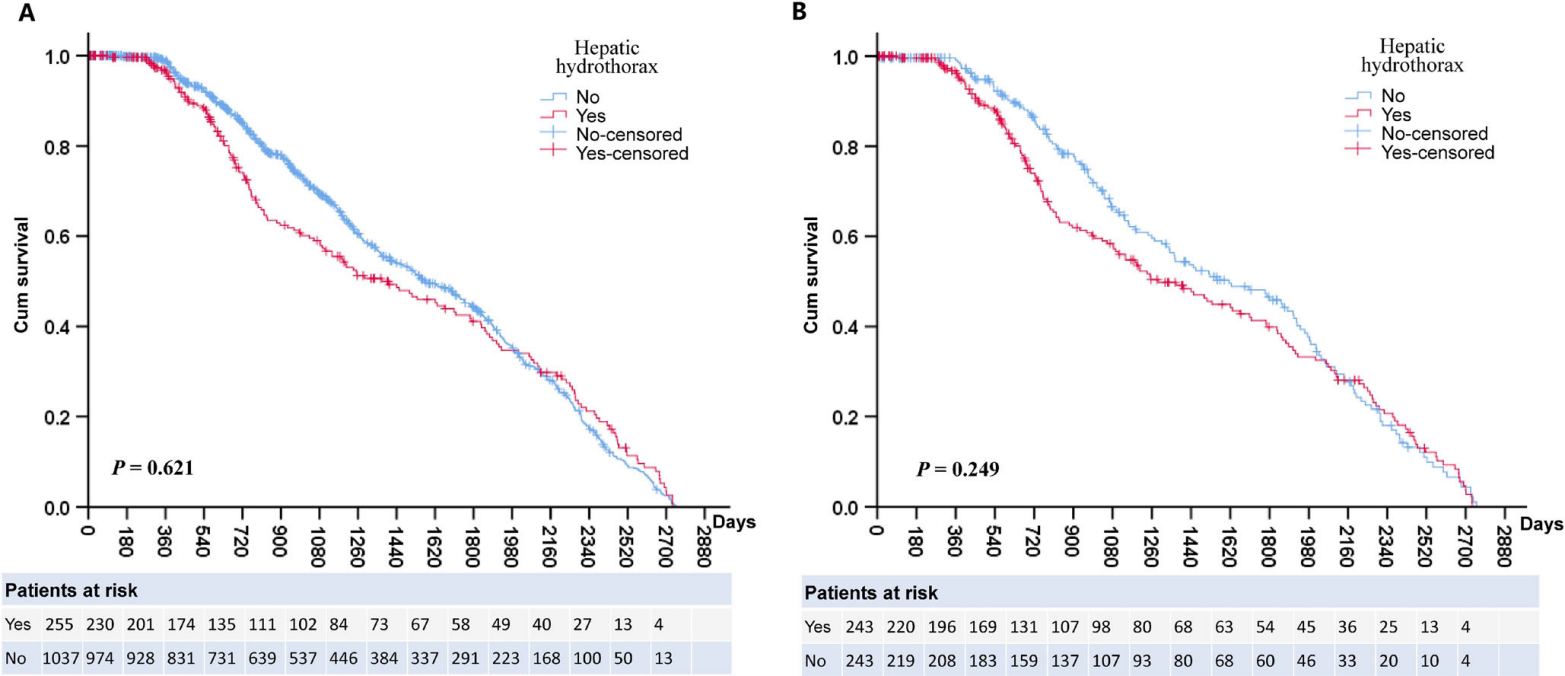
Kaplan–Meier curve of overall survival in all patients and in matched cohorts. **A** Kaplan–Meier curve showing that the overall survival of patients with hepatic hydrothorax (red line) was not different from that of patients without hepatic hydrothorax (blue line). **B** Kaplan–Meier curve showing that there was no significantly different cumulative survival between patients with hepatic hydrothorax (red line) and without hepatic hydrothorax (blue line) after propensity score matching. Statistical analysis: log-rank test

To evaluate the influence of HH on survival after TIPS in patients with cirrhosis, we compared survival between cirrhotic patients with and without HH by using the Kaplan–Meier survival estimate. The results showed that there was no difference in survival between the two groups (log-rank test, *p* = 0.621). The 1-year mortality rates were 12% and 8% in patients with and without HH, respectively (Fig. 2A). A Cox proportional hazards regression model was used to analyse the factors affecting survival. The results showed that hepatitis B virus infection and portal thrombosis over 50%, but not HH, increased the risk of death. Curve estimation analysis showed that age (model, cubic; adjusted *R*^2^ = 0.045, *p* < 0.001) and MELD score (model, cubic; adjusted *R*^2^ = 0.041, *p* < 0.001) were associated with death after TIPS (Table 4). To reduce the impact of baseline bias on patient survival, 255 patients with HH were matched to patients without HH at a 1:1 ratio by age, sex, aetiology, MELD score, main portal vein thrombosis, and splenectomy. Finally, 243 patients with HH were matched with 243 patients without HH with similar baseline characteristics (Supplementary Table 1). The cumulative survival in the HH group was not significantly different from that of the group without hydrothorax (log-rank test, *p* = 0.249) (Fig. 2B). The results of the Cox proportional hazards model showed that HH did not increase the mortality of TIPS in cirrhosis patients. Portal vein thrombosis over 50% independently predicted the mortality of cirrhosis patients treated with TIPS (HR, 1.386; 95% CI, 1.030–1.865; *p* = 0.031). Age and MELD were also associated with death of cirrhosis patients after TIPS (Table 5). These results suggest that although patients with HH have worse liver function, their mortality after TIPS does not increase.

**Table 4:** Analysis of survival-related factors

Univariate and multivariate Cox regression analysis						
Variable	Univariate analysis			Multivariate analysis		
	HR	95% CI	*p* value	HR	95% CI	*p* value
Gender (male)	0.842	0.733 0.967	**0.015**	0.928	0.798 1.079	0.330
Aetiology (HBV)	0.806	0.702 0.925	**0.002**	0.824	0.710 0.957	0.011^*^
PVT:MPV > 50%	1.412	1.211 1.648	**0.000**	1.480	1.253 1.747	0.000^*^
Splenectomy	1.007	0.824 1.229	0.949	0.844	0.681 1.046	0.121
Hepatic hydrothorax	1.044	0.878 1.242	0.626	1.035	0.870 1.231	0.702
Curve estimation						
		Models			Adjusted *R*^2^	*p* value
Age		Cubic			0.045	0.000^*^
MELD		Cubic			0.041	0.000^*^

*HBV* hepatitis B virus, *PVT* portal vein thrombosis, *MPV* main portal vein, *MELD* model of end-stage liver disease

The bold font and asterisks indicate a significant difference

**Table 5:** Analysis of survival-related factors in the matched cohort

Univariate and multivariate Cox regression analysis						
Variable	Univariate analysis			Multivariate analysis		
	HR	95% CI	*p* value	HR	95% CI	*p* value
Gender (male)	0.892	0.704 1.131	0.345	0.973	0.756 1.253	0.833
Aetiology (HBV)	0.836	0.661 1.057	0.135	0.853	0.667 1.091	0.206
PVT:MPV > 50%	1.361	1.035 1.789	0.028	1.386	1.030 1.865	0.031^*^
Splenectomy	1.046	0.730 1.497	0.807	0.874	0.5941 1.287	0.496
Hepatic hydrothorax	1.144	0.909 1.440	0.250	1.121	0.890 1.413	0.332
Curve estimation						
		Models			Adjusted *R*^2^	*p* value
Age		Logistic			0.049	0.000^*^
MELD		Cubic			0.028	0.003

*HBV* hepatitis B virus, *PVT* portal vein thrombosis, *MPV* main portal vein, *MELD* model of end-stage liver disease

The bold font and asterisks indicate a significant difference

### HH responds well to TIPS and does not increase the complications of TIPS

Cirrhosis patients with symptomatic HH respond well to TIPS. Among the 255 patients with HH, 3 were treated with TIPS because of refractory HH, while the remaining patients were treated with TIPS because of repeated variceal bleeding or refractory ascites. Nine patients died 1 month after TIPS. Of the 246 surviving patients, 96.7% (238/246) achieved a complete response, 2.8% (7/246) achieved a partial response, and 0.5% (1/246) had no response. At 6 months after TIPS, 25 patients had died. Of the 230 surviving patients, 99.5% (229/230) had a complete response and 0.5% (1/230) had a partial response.

HH did not increase the complications of TIPS. The incidence rates of stent stenosis and hepatic encephalopathy after TIPS in patients with HH were 13.3% (34/255) and 29.4% (75/255), respectively. The incidences of stent stenosis and hepatic encephalopathy were 15.4% (160/1037) and 26.4% (274/1037), respectively, in patients without HH after TIPS. There was no significant difference in the incidence of stent stenosis (13.3% vs. 15.4%, *p* = 0.705) or hepatic encephalopathy (29.4% vs. 26.4%, *p* = 0.928) between patients with and without HH.

## Discussion

HH is a decompensated event and a poor prognostic indicator in cirrhotic patients not treated with TIPS [12]. Patients with HH often have other decompensating events including ascites, and/or variceal haemorrhage [26]. For these patients, the effect of HH on survival after TIPS is unclear. Our study aimed to evaluate the influence of HH on the survival of cirrhosis patients after TIPS. In this study, 1292 cirrhosis patients with portal hypertension complications treated with TIPS were enrolled. Among those, 255 patients had HH. No significant difference in survival was found between patients with and without HH after TIPS. Then, 243 patients with HH were matched with 243 patients without HH. There was also no significant difference in overall survival between patients with HH and those without HH after TIPS. Univariate and multivariate analyses showed that main portal vein thrombosis (> 50%), but not HH, was a prognostic factor of long-term survival for TIPS in cirrhosis patients. Our study indicated that concomitant HH did not increase the risk of death of TIPS. Our findings provide a basis for TIPS treatment in cirrhosis patients with TIPS indications accompanied by HH.

This study found no difference in survival time after TIPS between patients with and without HH. Previous studies have shown that among those who have not undergone TIPS, patients with HH have a higher risk of death than those without HH, even when their liver function was similar [8, 12]. Why is there no difference in survival after TIPS? It may be because TIPS reduces the risk of death in patients with HH. The study of Osman KT et al showed that the survival of cirrhosis patients with refractory hydrothorax who did not receive TIPS was worse than that of patients with refractory ascites [12]. Another study showed no significant difference in overall survival after TIPS between patients with and without refractory HH [27]. The results of these two studies indirectly indicated that TIPS reduced the risk of death in patients with refractory HH. These results are consistent with our findings. The risk of death was higher in patients with HH than in those without HH, which was the conclusion of those who did not receive TIPS. Our study found no difference in the risk of death between the two groups after TIPS treatment.

A meta-analysis showed that the 1-year mortality rate in patients with refractory HH receiving TIPS was as high as 50% [16]. However, the 1-year mortality rate in patients with HH receiving TIPS was only 12% in our study. One reason for the inconsistent results may be the different timing of TIPS application. We used TIPS at the time patients had not yet developed refractory HH. Of course, the indications for TIPS in these patients are not HH, but they are accompanied by HH. The early use of TIPS can improve the survival of high-risk patients with cirrhosis complications. For example, early TIPS can improve survival in high-risk patients with acute bleeding [21], as well as ascites [19, 20, 28, 29]. Therefore, the early application of TIPS in the treatment of HH is also worth exploring. Previous studies have shown that patients with HH under 60 years old with good liver function respond well to TIPS treatment and have a high survival rate [15, 17, 30]. These results support the early use of TIPS for HH. However, the optimal timing for TIPS intervention requires large, multicentre prospective studies.

Previous studies found that poor liver function was a predictor of death in patients with refractory HH treated with TIPS [15, 31]. While we found that patients with HH had worse liver function, their mortality after TIPS did not increase in this study. The effect of liver function on the long-term survival of patients with HH treated with TIPS was not consistent with previous studies. This was mainly because the object of the previous studies was refractory HH, while the object of our study was nonrefractory HH. Most of the patients with HH in our study had an earlier course of decompensated liver cirrhosis and had not reached the refractory HH stage. Therefore, although nonrefractory HH was associated with worse liver function, it did not increase the mortality of TIPS. Of course, further studies are needed to confirm this.

Our study had the following limitations: First, this was a retrospective study. Second, the main clinical problem in most patients was not HH but other decompensated events accompanied by HH. Therefore, it was impossible to evaluate the response of HH to diuretic and/or thoracocentesis treatment, and it was difficult to accurately distinguish the stage of HH. Third, although we matched age, sex, MELD score, and other clinical characteristics between patients with and without HH, rigorous prospective controlled studies are needed to validate our findings.

In conclusion, our study suggested that, when patients with decompensated events are indicated for TIPS, the presence of HH should not affect the choice of TIPS treatment, since there was no increased risk of death after TIPS compared with patients without HH.

## Supplementary Information

ESM 1 (DOCX 61 kb)

## Declarations

### Guarantor

The scientific guarantor of this publication is Tie Jun.

### Statistics and biometry

No complex statistical methods were necessary for this paper.

### Methodology

• retrospective

• case–control study

## Abbreviations

HBV: Hepatitis B virus
HE: Hepatic encephalopathy
HH: Hepatic hydrothorax
MELD: Model of end-stage liver disease
MPV: Main portal vein
PPG: Portal pressure gradient
PVT: Portal vein thrombosis
TIPS: Transjugular intrahepatic portosystemic shunt
