# Supplementary material for: Hepatic hydrothorax does not increase the risk of death after transjugular intrahepatic portosystemic shunt in cirrhosis patients
Source: Eur Radiol. 2022 Dec 28;33(5):3407–15. doi: 10.1007/s00330-022-09357-3 (PMC10121519; doi:10.1007/s00330-022-09357-3)
Supplement: Supplementary file 1 — (DOCX 61 kb) [file 330_2022_9357_MOESM1_ESM.docx]

**Supplementary methods**

The response of hepatic hydrothorax (HH) to TIPS was evaluated according to both clinical symptoms and radiographic evidence. If the evaluations were inconsistent, they were calculated according to the poor evaluation result. The response of HH to TIPS was categorized as complete, partial, or absent. The evaluation based on clinical symptoms was as follows: (1) Complete response was defined as the resolution of shortness of breath, and no longer requiring thoracentesis. (2) Partial response was defined as improvement of shortness of breath but without complete symptomatic resolution, and thoracentesis being required less frequently than pre-TIPSS. (3) An absent response was defined as persistent or worsening symptoms of shortness of breath and/or a persistent need for thoracentesis. The evaluation based on radiographic evidence was as follows: (1) Complete response was defined as undetectable pleural effusion on chest X-ray, CT, or ultrasonogram; (2) Partial response was defined as a more than 50% reduction of pleural effusion compared to pre-TIPS. The quantification of hepatic hydrothorax was performed using picture archiving and communication system (PACS)-based volumetric tools. A 50% reduction represented a 50% reduction in pleural effusion volume measured by PACS after TIPS compared to the preoperative baseline. These methods were further elaborated in the revised manuscript. (3) An absent response was defined as a less than 50% reduction or increase in pleural effusion. This study used either clinical and/or radiologic criteria to assess the response to TIPS.

**Supplementary tables**

**Supplementary table 1.** **Baseline characteristics of the cohort after propensity score matching**

| **Parameter**  Median (range) or absolute (percentage) | **HH (243)** | **No HH (243)** | **P Value** |
| --- | --- | --- | --- |
| Age (years) | 52 (26-85) | 54 (20-79) | 0.364 |
| Gender |  |  |  |
| Male | 137 (56.4%) | 151 (62.1%) | 0.196 |
| Female | 106 (43.6%) | 92 (37.9%) |  |
| Aetiology |  |  |  |
| HBV | 141 (58.0%) | 143 (58.8%) | 0.854 |
| Others | 102 (42.0%) | 100 (41.2%) |  |
| Manifestations |  |  |  |
| Variceal bleeding | 208 (85.6%) | 214 (88.1%) | 0.421 |
| Refractory ascites | 30 (12.4%) | 28 (11.5%) | 0.780 |
| Refractory HH | 3 (1.2%) | 0 (0%) | 0.082 |
| others | 2 (0.8%) | 1 (0.4%) | 0.562 |
| MELD | 12 (0-27) | 12 (1-28) | 0.519 |
| PVT: MPV > 50% | 66 (27.2%) | 58 (23.9%) | 0.405 |
| Splenectomy | 34 (14.0%) | 23 (9.5%) | 0.121 |

HBV, hepatitis B virus; HH, hepatic hydrothorax; MELD, model of end-stage liver disease; PVT, portal vein thrombosis; MPV, main portal vein.
